# A robust MRI-compatible system to facilitate highly accurate stereotactic administration of therapeutic agents to targets within the brain of a large animal model

**DOI:** 10.1016/j.jneumeth.2010.10.023

**Published:** 2011-01-30

**Authors:** E. White, M. Woolley, A. Bienemann, D.E. Johnson, M. Wyatt, G. Murray, H. Taylor, S.S. Gill

**Affiliations:** a Department of Neurosurgery, Frenchay Hospital, Bristol, BS16 1LE UK; b Functional Neurosurgery Research Group, Institute of Clinical Neurosciences, School of Clinical Sciences, Bristol University, AMBI LABS, Southmead Hospital, Bristol, BS10 5NB, UK

**Keywords:** Convection-enhanced delivery, Pig model, Stereotactic delivery, Viral vector

## Abstract

Achieving accurate intracranial electrode or catheter placement is critical in clinical practice in order to maximise the efficacy of deep brain stimulation and drug delivery respectively as well as to minimise side-effects. We have developed a highly accurate and robust method for MRI-guided, stereotactic delivery of catheters and electrodes to deep target structures in the brain of pigs. This study outlines the development of this equipment and animal model. Specifically this system enables reliable head immobilisation, acquisition of high-resolution MR images, precise co-registration of MRI and stereotactic spaces and overall rigidity to facilitate accurate burr hole-generation and catheter implantation.

To demonstrate the utility of this system, in this study a total of twelve catheters were implanted into the putamen of six Large White Landrace pigs. All implants were accurately placed into the putamen. Target accuracy had a mean Euclidean distance of 0.623 mm (standard deviation of 0.33 mm). This method has allowed us to accurately insert fine cannulae, suitable for the administration of therapeutic agents by convection-enhanced delivery (CED), into the brain of pigs. This study provides summary evidence of a robust system for catheter implantation into the brain of a large animal model. We are currently using this stereotactic system, implantation procedure and animal model to develop catheter-based drug delivery systems that will be translated into human clinical trials, as well as to model the distribution of therapeutic agents administered by CED over large volumes of brain.

## Research highlights

▶ Development of a highly accurate and robust method for MRI-guided, stereotactic delivery of catheters into the brain of pigs. ▶ Reliable head immobilisation, acquisition of high-resolution MR images, precise co-registration of MRI and stereotactic spaces to facilitate accurate burr hole-generation and catheter implantation. ▶ Implants were accurately placed into the putamen with a mean Euclidean distance of 0.623 mm (standard deviation of 0.33 mm).

## 1 Introduction

The use of pigs in neurological research offers significant advantages over the use of other animal models. Specifically, the use of pigs is less expensive and generates fewer ethical concerns than the use of primates, particularly when subtle behavioural outcome measurements are not required. Due to the comparatively large size and gyrencephalic structure of pig brain compared to other animals commonly used in medical research such as mice, rats and rabbits, which have small lissencephalic (smooth) brains, the pig is an ideal model to develop novel neurosurgical techniques that can be directly translated into human clinical trials. In particular, the large size of the pig brain facilitates high-resolution imaging in standard clinical MRI scanners, the use of human stereotactic systems and the implantation of human scale equipment into the brain (e.g. deep brain stimulation electrodes or drug delivery cannulae). Indeed, Bjarkam et al. have previously developed a stereotactic procedure for the implantation of electrodes into the basal ganglia of Gottingen minipigs (Bjarkam et al., 2004, 2004), and Saito et al. and Sakumoto et al. have developed a technique for the implantation of catheters into the third ventricle of Chinese Meishan pigs (Saito et al., 1998; Sakumoto et al., 2004).

Convection-enhanced delivery (CED) is a rationale technique for the direct intracranial administration of therapeutic agents into the brain. CED necessitates the use of an appropriate catheter and infusion flow-rate to distribute therapeutic agents by bulk-flow directly into the brain extracellular space (Bobo et al., 1994; Morrison et al., 1994). At present, there is no commercially available device that is compatible with CED and suitable for the direct intracranial administration of viral vectors into the brain in clinical practice. Previous clinical trials have utilised a number of different delivery techniques. For example, gene therapy trials for the treatment of high-grade gliomas have employed either stereotactic, or free-hand injection (Chiocca et al., 2004; Germano et al., 2003; Harrow et al., 2004; Immonen et al., 2004; Markert et al., 2000; Papanastassiou et al., 2002; Rampling et al., 2000) using unspecified needles and syringes or an infusion through an intra-tumourally implanted ventricular catheter (Lang et al., 2003). Similarly, gene therapy trials for Parkinson's disease (Eberling et al., 2008; Kaplitt et al., 2007; Marks et al., 2008); Canavan's disease (McPhee et al., 2006) and late infantile neuronal ceroid lipofuscinosis (Worgall et al., 2008) have employed untested delivery systems, developed in-house.

To overcome the lack of validated catheter systems for the delivery of therapeutic agents to the brain and to bridge the gap between small animal and human studies, we have developed a pig model for the development and testing of stereotactically delivered catheters that are compatible with CED and can be utilised to achieve widespread distribution of a range of therapeutic agents.

In contrast to a number of earlier studies undertaken to develop stereotactic procedures in pigs (Bjarkam et al., 2004, 2009; Rosendal et al., 2009; Sakumoto et al., 2004), we elected to use crossbred Large White/Landrace pigs rather than minipigs for this study for a number of reasons. Most importantly, as CED is capable of achieving drug distribution over large volumes of brain it was essential to test drug delivery devices within an animal model with a brain as large as possible and the available literature demonstrates that crossbred agricultural pigs such as the Large White/Landrace pig have a significantly larger brain than mature Gottingen minipigs (Jelsing et al., 2006). Furthermore, the large frontal air sinus in Gottingen minipigs can compromise MR imaging as well as complicate intracranial implantation of electrodes and catheters.

The principal purpose of this study was to develop a large animal model in which catheters and insertion techniques could be developed and translated into human trials without significant modifications. The development of this model required a number of problems to be tackled and compromises made. These included:

1. What age and weight of pig to use?
2. How to immobilise the pig head, so that MRI and stereotactic spaces could be accurately imaged and co-registered?
3. Which stereotactic frame to use and how to modify this for cannula implantation in pigs?
4. How to deliver cannulae accurately to deep brain targets?
5. How to quantitatively assess cannula implantation accuracy?

The approaches employed to tackle these problems are outlined in the Materials and Methods, and is followed in Section 3 by a study undertaken to demonstrate the utility of this animal model and stereotactic technique. To date, we have used this animal model and stereotactic equipment to develop a cannula for acute intracranial drug delivery as well as an implantable catheter system for chronic and intermittent drug delivery. Both of these drug delivery systems are compatible with CED, and it is our intention to use them in forthcoming clinical trials.

## 2 Materials and methods

### 2.1 Sedation and anaesthesia

A total of six 45 kg, male Large White Landrace pigs were used in this study.

Animal studies were conducted in accordance with the UK Scientific Procedures Act (1986) under appropriate project and personal licences. Animals were sedated with intramuscular ketamine (10 mg/kg), intubated and subsequently anesthetised with a 1.5–5% isoflurane.

### 2.2 Head immobilisation and brain imaging

Felix et al. developed a stereotactic atlas of the Large White Landrace pig brain (Felix et al., 1999). However, due to subtle intrinsic variability in anatomy between animals and the inherent distortion of this histological atlas, it was not feasible to use this atlas for precise cannula implantation *in vivo*. Consequently we developed an MRI-targeted stereotactic approach for these studies using an MRI scanner with a field strength of 1.5 T (Intera, Philips, UK).

Radiographic examination of a Large White Landrace head revealed a number of bony structures into which MRI-compatible screws could be inserted to achieve head fixation. These structures included the zygoma bilaterally and regions of thick bone in the frontal and occipital bones. In the course of these experiments a number of conceptual designs were evaluated. For the sake of brevity an overview of the main devices follows.

**Fig. 1:**
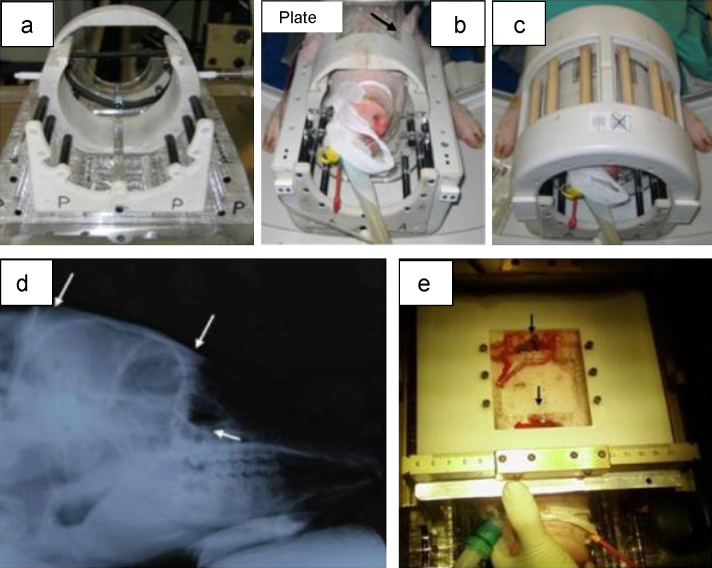
Initial methods for pig head immobilisation and MR imaging: (a) Posterior aspect of head fixation device, incorporating snout-strap and zygomatic screws, and designed to fit into a knee coil. (b) Image of immobilised porcine head, with fixated Perspex head plate, located in an open knee coil. (c) Image of pig immobilised in head fixation device locked in a closed Sence knee coil. (d) Lateral radiographic image of a porcine head, illustrating the points of potential screw-fixation to the frontal and occipital bones, and zygoma are arrowed. (e) Peri-operative image, illustrating anterior view of skull-fixation plate attached to a modified Leksell D stereotactic frame. The site of bone screws into the frontal and occipital bones are arrowed.

Initially, an open-topped, head-fixation tube, incorporating bilateral titanium-tipped zygomatic screws and a Velcro snout-strap was developed to fit within a 1.5 T Sense knee coil (Fig. 1a–c). On the superior aspect of this tube, an N-shaped fiducial plate could be attached during imaging. This initial design proved inadequate, as it was technically difficult to accurately insert the zygomatic screws into the correct position whilst the animal was in the tube due to a lack of space. Consequently an alternative method to zygomatic fixation was developed, involving the use of a flat Perspex plate attached on top of the skull (Fig. 1b: arrowed) via titanium screws inserted into the frontal and occipital bones (Fig. 1d and e). This head-fixation plate was also designed for use with the Sense knee coil and incorporated a central aperture, into which the N-shaped fiducial could be attached during imaging and had the added advantage that it could be fitted to the top of a stereotactic frame, making it easier to access the surgical field (Fig. 1e).

**Fig. 2:**
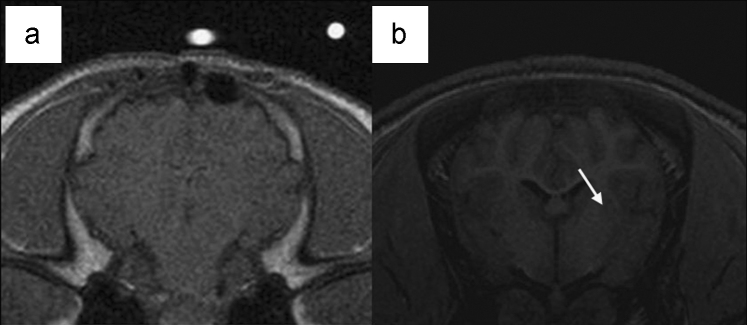
T1-weighted coronal pig brain images of a 3-month old pig (a) and a 5-month old pig (b). Note that the lentiform nucleus is clearly visible at 5 months of age (arrowed).

The critical limitation of this approach was found to be the single anterior fixing screw in the frontal bone that occasionally failed to provide a means of robust support due to the spongy nature of this bone in animals of this age (3 months). Indeed the use of the Sense knee coil was restricted to pigs of no more than 3 months of age due to the extremely limited amount of space inside these coils (Fig. 1b). Furthermore, the use of such young pigs was detrimental to the image quality that could be achieved. Specifically, in contrast to MR imaging of 3-month old pigs, T1-weighted MR imaging of 4–5-month old pigs led to improvements in grey–white differentiation that made clear identification of the basal ganglia possible (Fig. 2: lentiform nucleus arrowed).

As white matter myelination is known to occur in the early post-natal period and be completed by 6 months of age in minipigs (Fang et al., 2005) we hypothesised that if older Large White Landrace pigs could be effectively immobilised in a head fixation device, which provided the space and flexibility for use with Sense Flex-L coils, then better imaging, as defined by improvements in grey–white matter differentiation, could be achieved. Therefore a more robust device for head immobilisation was developed based on that presented by Bjarkam et al., despite this set-up inevitably increasing the distance between the coils and the brain (Bjarkam et al., 2004). However, due to the larger head size of Large White/Landrace pigs and significant variability in head shapes between animals, significant modifications were required.

**Fig. 3:**
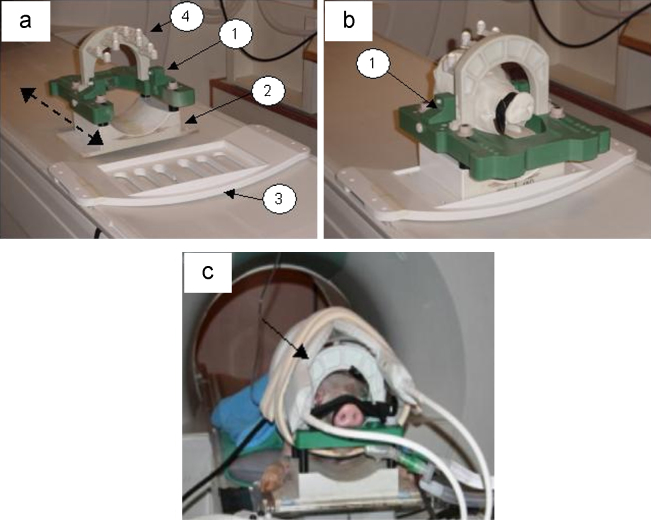
Final method for pig head immobilisation and MR imaging: (a) Parts of the MRI compatible pig head fixation and MRI localisation device; head fixation frame (1), lower mounting unit (2), MRI bed location unit (3) and MRI visible fiducial arc (4). (b) Pig head fixation device and 3D model of pig head illustrating zygomatic screw housing (1). (c) Large White Landrace pig model (≈45 kg) on MRI scanner bed. Head immobilised with Flex-L coils (arrowed) attached and encompassing fiducial arc and pig head for pre- and post-operative T1-weighted imaging.

This head fixation device was designed to accommodate Large White Landrace pigs weighing up to 75 kg (approximately 6 months of age). The frame (illustrated in Fig. 3a) was machined from a hard grade of polyurethane (Huntsman BM 5172), which is light in weight, possesses high stiffness and is MRI inert. Slideways were mounted on top of four acetyl pillars located on the lower mounting unit. These provide lateral adjustment for head fixation (Fig. 3a and b). The head frame accommodated two interchangeable zygomatic screw housings, possessing opposing lateral adjustment (Fig. 3b) to the frame in order to provide immobilisation for pigs with variable head geometry. Head immobilisation was then augmented using a mouldable palate tray to which a Velcro snout strap was attached. In addition, a fiducial arc could be accurately positioned via specific location dowels in the frame. Animals could then be transported to the MRI scanner where the head immobilisation device could be positioned in its bed location unit. This unit provided a means for repeatable positioning for pre- and post-operative imaging, which was essential for target attainment verification following stereotactic cannula implantation. Following head immobilisation, Flex-L coils could rapidly and securely be attached laterally around the fiducial arc and the pig head (arrowed in Fig. 3c). In contrast to the head-fixation device developed by Bjarkam et al., this system device allowed fixation of pig heads of variable size and shape, incorporated a mouldable pallet-tray, facilitated high-resolution imaging using flexible MRI-coils and allowed simple and reproducible placement of fiducials close to the brain (Bjarkam et al., 2004). Head-fixation and fiducial placement with this equipment took no longer than 10 min.

Fig. 3 illustrates the various component parts of the final pig head fixation device and MRI fiducial arc. As anticipated this set-up produced high quality images in which grey–white tissue interfaces were clearly visible, rendering stereotactic implantation of cannulae into deep grey matter nuclei technically feasible.

### 2.3 Stereotactic system

**Fig. 4:**
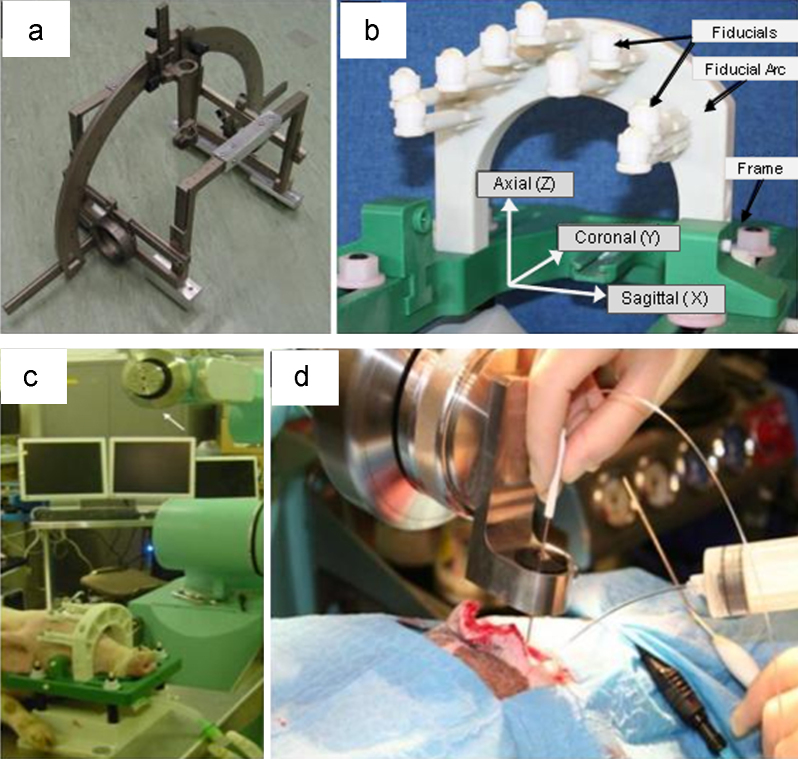
Stereotactic methods and co-registration for target attainment: (a) Image of Leksell D stereotactic frame. (b) Final head-fixation frame with fiducial arc and MRI visible balls. (c) Image of immobilised pig under the Pathfinder surgical robotic arm: end effector location is arrowed. Note the replacement of optical reflector balls around the fiducial arc to aid robot/head-frame co-registration. (d) Image illustrating robot end effector used for the delivery of stiff cannula to targets in the brain.

Initially a Leksell D stereotactic frame was employed for the stereotactic insertion of cannulae into pig brain *in vivo* (Fig. 4a). In-house software was developed, which co-registered the MRI and stereotactic volumes, based on a standard spatial relationship between the MRI fiducials and the standard frame position when it was attached to the pig head fixation device (Fig. 1e). Collets were designed to fit into the stereoguide and accommodate a range of drills and tools designed for burr hole formation and cannula insertion respectively. The use of this frame was however time-consuming and the inherent instability of the frame's arc led to difficulties drilling very precisely formed burr holes. Consequently the frame was replaced by a commercially available surgical robotic arm (Pathfinder, Prosurgics, UK) and surgical planning software (Mayfield ACCISS-II, UK). This robotic stereotactic arm had the key advantage of providing an extremely solid base for burr hole drilling, leading to the formation of a perfectly round burr hole into which a cannula hub would push-fit.

Once the pig was immobilised in the head fixation device, nine MRI fiducial spheres were attached to fixed, pre-determined locations on the fiducial arc (Fig. 4b and Table 1). This fiducial arc was then secured via specific positioning dowels to the head frame. The pig could then be transported to the MRI scanner, where it would undergo pre-operative MR imaging to obtain contiguous T1-weighted coronal slices (1 mm slice thickness) of a volume, which included the fiducials and brain. For the T1 imaging the parameters are as follows: FOV AP 200 mm:RL 159 mm:FH72 mm, voxel size: AP 0.575 mm:RL 0.575 mm:FH 0.8 mm; matrix size: M × P 378 × 277.

### 2.4 Surgical procedures and contrast infusions

Stereotactic insertion of cannulae was achieved using a series of end-effectors, designed to accommodate specific drills for burr hole generation and tools for cannula insertion, which were attached to the robotic arm (Fig. 4c). As the distance from the target position to the robotic stereotactic arm was known, accurate stereotactic delivery of cannula to specific areas in the brain could be achieved.

Surgical planning was performed using ACCISS-II planning software to determine target locations, cannula entry points and trajectories. Specifically, target location and cannula trajectory were identified by the surgeon from the pre-operative T1 MR images such that the cannula tip would be approximately in the middle of the putamen and the cannula would not traverse a sulcus or large vessel. Having identified the locations of cannula entry on the animal's head, a U-shaped skin flap was raised and the skull surface exposed. Burr-holes with a diameter of 8 mm were then drilled using a series of hand-drills inserted through the stereoguide. A catheter system designed in-house was then implanted. Briefly, this catheter system, which will be the subject of a forthcoming paper submission, was constructed from a 0.4 mm outer diameter carbothane tube inserted through a 0.6 mm carbothane guide-tube. This guide-tube was inserted through a carbothane hub implanted into the 8 mm burr hole.

Infusions of 120 μl of Gadolinium-DTPA (Magnevist: Bayer Healthcare, Germany) mixed in sterile 0.9% sterile saline to a concentration of 0.25% were then undertaken using the following infusion regime: 0.5 μl/min for 5 min, 1 μl/min for 5 min, 2.5 μl/min for 5 min and then 5 μl/min for 20 min. The wound was closed with interrupted 4/0 prolene sutures. The animal was then taken out of the head fixation device and the zygomatic wounds closed with interrupted 4/0 prolene sutures. The animal was then woken from general anaesthesia, extubated and allowed to recover in isolation for 24 h. Animals were inspected twice daily during the recovery period for signs of wound infection or breakdown, neurological deficits or abnormal behaviour.

### 2.5 Quantitative target accuracy verification

**Fig. 5:**
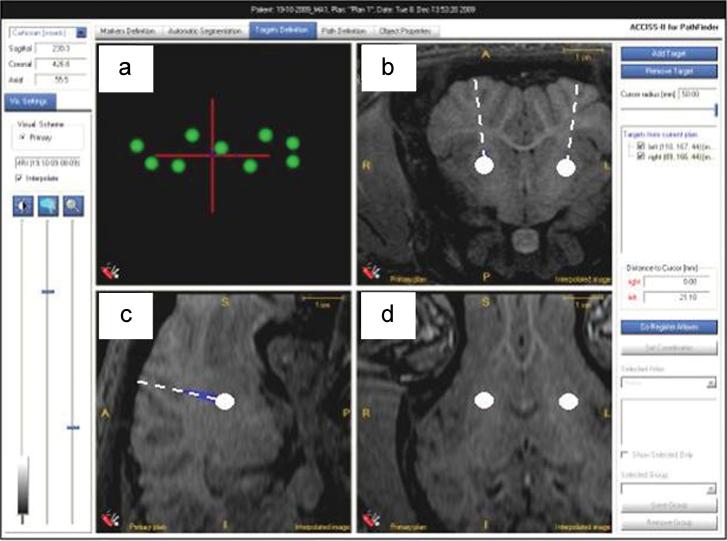
ACCISS-II screenshot of a typical pre-operative implantation plan: (a) Axial view of fiducial spheres. (b) Coronal image illustrating bilateral planned targets and trajectories into the putamen. (c) Sagittal interpolated image illustrating a single target and trajectory. (d) Axial interpolated image illustrating the bilateral targets in the putamen.

Compared to stereotactic surgical planning using a brain atlas, subject-specific image-guided surgical planning has the advantage that one can identify anatomical target points with certainty, whilst ensuring implant trajectories avoid important brain structure and vital blood vessels. In practice we found it necessary to insert cannula approximately perpendicular to the skull facilitate cannula fixation to the skull surface. A plan for bilateral infusions into the putamen of a 45 kg, male Large White Landrace pig is illustrated in Fig. 5. Using ACCISS-II planning software only the coronal images were used. Sagittal and axial images were derived by the software from the original coronal images. They were used to aid clinical judgement in ensuring a perpendicular approach was employed and that obvious blood vessels were avoided.

**Fig. 6:**
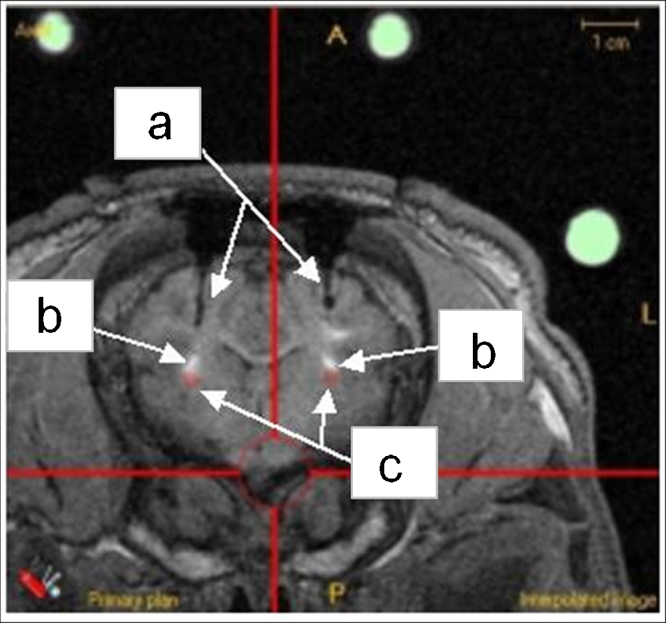
ACCISS-II image of a typical post-operative images of contrast distribution following bilateral cannula implantation: (a) Bilateral cannula tracks. (b) Infused contrast (0.25% gadolinium/saline mix). (c) Cannula-tip locations.

Once the implant procedure was complete, a post-operative MRI scan was obtained to evaluate cannula location. In all cases, the cannula-tip location was clearly visible as a very small volume of hypointensity. The centre of this volume was arbitrarily defined as the cannula-tip location. As the MRI bed-location unit (Fig. 3) was designed to locate each pig, including the fiducial arc, in the same position within the MRI scanner for pre- and post-operative MR imaging, it was possible to exactly identify the planned target co-ordinates and cannula trajectory in the post-operative scan. Pre- and post-operative scans were then superimposed, showing the planned cannula location on to the actual cannula position in the post-operative image. This was performed using the ACCISS II software (Fig. 6). A quantitative assessment of the difference between the planned catheter tip location and the centre of the post-operative infusion was then performed in all three planes (coronal [*y*], sagittal [*x*] and axial [*z*]) using the ruler facility in ACCISS-II. Planned target trajectories, were also superimposed to aid in the assessment of the level of cannula displacement.

### 2.6 Relative fiducial co-ordinates and calculation of re-positional error

The fiducial positions are identified in ACCISS-II using a built in automatic search routine. A Matlab program was devised to extract the fiducial co-ordinates, target co-ordinates and entry points, if present, from the ACCISS-II files, for both the pre- and post-operative scans. The Matlab program then computed the transformation necessary to map the fiducials from the pre-operative scan space, to the post-operative scan space.

Since the transformation between the two Cartesian co-ordinate systems of the scans can be thought of as the result of a rigid body motion, the transformation can be decomposed into a rotation and translation. Three degrees-of-freedom are associated with translation. Three degrees-of-freedom are associated with rotation. A further degree-of-freedom if associated with scaling.

Since it would not be expected to be able to find a single transformation which perfectly maps the co-ordinates of the fiducials from the pre-operative scan to co-ordinates of the fiducials in the post-operative scan, a method is needed to obtain the best match.

Here a closed-form solution to the least squared residuals is used – one which does not require iteration. The method is described by Horn and its implementation in Matlab has been described by Wengert (Horn, 1987; Wengert, 2008). This closed form solution provides in a single step the best possible transformation given the co-ordinates of the fiducials in the two co-ordinate systems (pre- and post-operative scan space). The method uses unit quaternions to represent the rotations.

The difference between the measured pre-operative fiducial co-ordinate position and the computed post-operative fiducial co-ordinate position (Euclidean distance) gives a measure of accuracy for fiducial location provided by the following formula:

$$e^{2}=\sum_{1}^{9}\Delta x^{2}+\Delta y^{2}+\Delta z^{2}$$

**Fig. 7:**
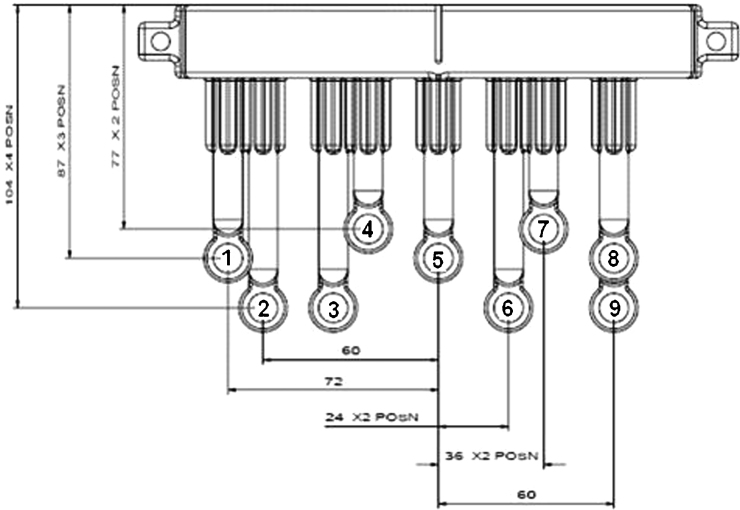
Sagittal (*x*) and coronal (*y*) co-ordinates (mm) for fiducials relative to supporting arc (numbered 1–9).

Here it was found that the average re-positional error ($\sqrt{e}$) over 30 positional trials to be 0.16 mm. A minimum of three points (fiducials) in both Cartesian co-ordinate systems (pre- and post-operative), provide nine constraints (three co-ordinates each: *x*, *y* and *z* or sagittal, coronal and axial), which is a sufficient quantity to determine the unknowns (providing that the three points are not co-linear). However, although the fiducials were completely spherical and the voxels had equal dimensions in *X*, *Y* and *Z*, due to partial voluming, they often appeared slightly oval when imaged by MRI. This introduced a degree of error into measurements of fiducial positions determined by the ACCISS-II software. Therefore, in practice, greater accuracy is achieved by increasing the quantity of points. The use of nine fiducials in our study (located as identified in Fig. 7) seemed to provide adequate co-registration accuracy.

The input parameters for the Matlab function, as presented by Wengert are the two sets of fiducial co-ordinates from the pre- (*F*_1_) and post-operative (*F*_2_) scans (Wengert, 2008). The output parameters are a 3 × 3 rotation matrix (*r*), a 3 × 1 translation vector (*t*), a scaling factor *s*, and the residual error *e*. We base our transformation on a special affine transformation equation, as discussed in Maintz (1998). An affine or rigid transformation can be described using a single constant matrix (a) using equation: *y*_*i*_ = *a*_*ij*_*x*_*j*_ where, in our case, *x* and *y* are the pre- and post-operative co-ordinate vectors. In the affine case the equation is constrained as described below:

**Figure:**
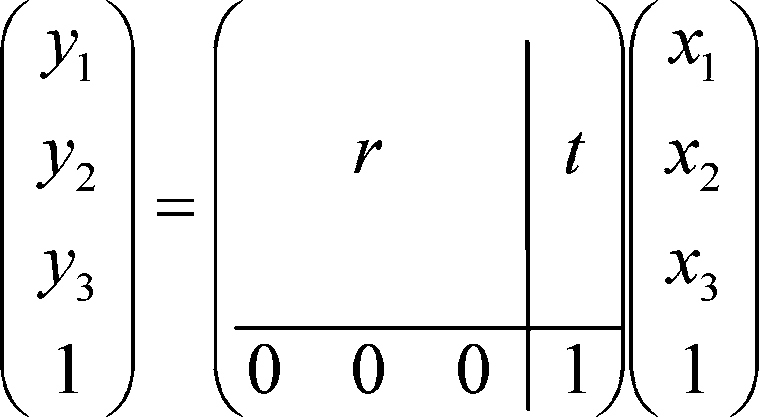

where *t* is the arbatory 3 × 1 translation vector, and *r* is the 3 × 3 rotation matrix defined by:

$$r_{il}=r_{ij}^{\left(1\right)}r_{jk}^{\left(2\right)}r_{kl}^{\left(3\right)}$$

Expanding *r* gives:

$$r=\left[\begin{array}{ccc} \cos\;\alpha \;\cos\;\beta & -\cos\;\alpha \;\sin\;\beta \;\cos\;\gamma +\sin\;\alpha \;\sin\;\gamma & \cos\;\alpha \;\sin\;\beta \;\sin\;\gamma +\sin\;\alpha \;\cos\;\gamma \\ \sin\;\beta & \cos\;\beta \;\cos\;\gamma & -\cos\;\beta \sin\;\gamma \\ -\sin\;\alpha \;\cos\beta & \sin\;\alpha \;\sin\;\beta \;\cos\;\gamma +\cos\;\alpha \;\sin\;\gamma & -\sin\;\alpha \;\sin\;\beta \;\sin\;\gamma +\cos\;\alpha \;\cos\;\gamma \end{array}\right]$$

**Fig. 8:**
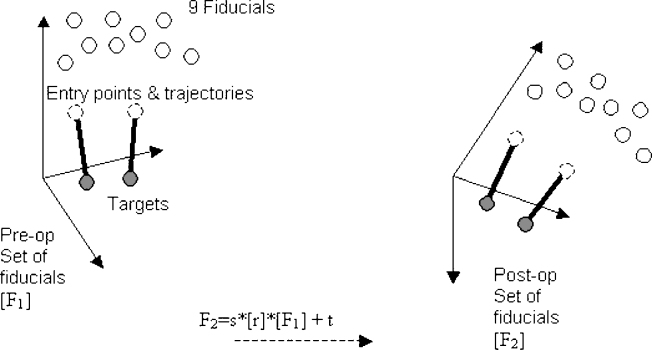
Schematic representation of pre- and post-operative fiducials in different Cartesian co-ordinate systems.

Fig. 8 illustrates the relationship between the pre- (*F*_1_) and post-operative (*F*_2_) fiducials in different Cartesian co-ordinate systems. Finally, the transformation between the two sets of fiducials *F*_1_ and *F*_2_ are shown as rotation matrix [*r*] and translation matrix [*t*] such that:

$$[F_{2}]=s*[r]*[F_{1}]+t\text{.}$$

## 3 Results

### 3.1 Assessment of target accuracy

**Fig. 9:**
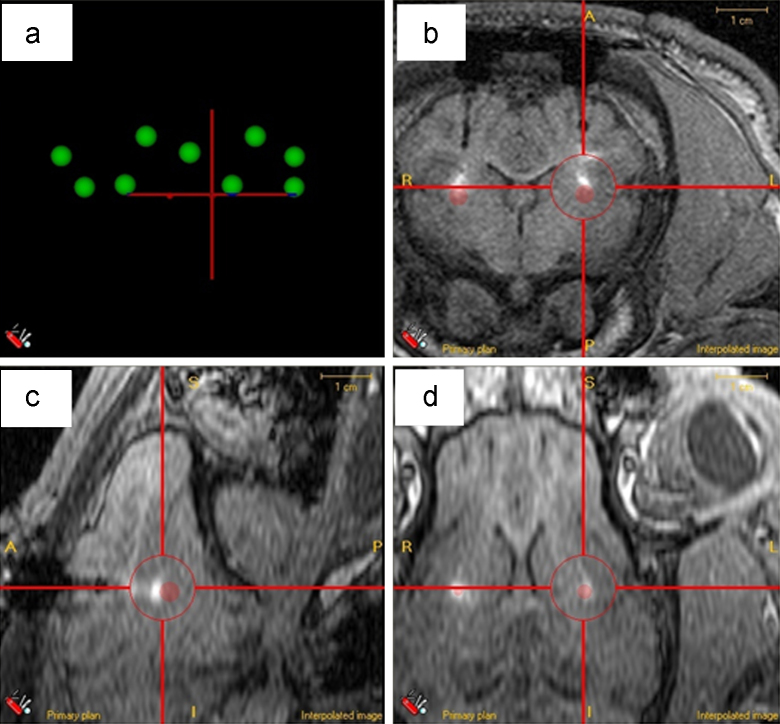
ACCISS-II illustrating post-operative bilateral infusions with original pre-operative ‘planned targets’ suitably transposed. (a) Fiducial sphere arrangement in axial (plan) view. (b) Coronal slice illustrating infusate delivery at target sites (putamen) in both hemispheres. Circular ‘sphere’ indicates planned target point. (c) Same MRI slice in saggital section illustrating infusate delivery at target sights (putamen) in both hemispheres. Circular ‘dot’ indicates planned target point. (d) Same MRI slice in axial section illustrating infusate delivery at target sights (putamen) in both hemispheres. Circular ‘dot’ indicates planned target point.

Fig. 9 illustrates superimposed, planned and actual target positions from one of the six pigs used in the implant study. Fig. 9b–d demonstrates the targets in all three planes (coronal, sagittal and axial). T1-weighted MR imaging of acute infusions of 120 μl of Gadolinium-DTPA (Magnevist), demonstrated widespread distribution in the putamen in both left and right hemisphere.

Intended Cartesian co-ordinate target positions for both hemispheres for all three planes, taken from ACCISS II are listed in Table 2. The measured differences between the centre of the superimposed original target point and the catheter tip (indicated by the centre of the infusion) is also listed along with the calculated Euclidean error distance. In summary, mean cannula implantation inaccuracy was 0.623 mm. This demonstrates that this stereotactic procedure is compatible with highly accurate cannula implantation into deep structures within pig brain.

## 4 Discussion

Minipigs have been bred extensively for use in medical research. Their small size and subsequent ease of handling makes them suitable for long-term neurological studies. Unfortunately the use of Gottingen minipigs for intracranial implantation procedures in the UK is not straightforward. Firstly, although the skull thickness of this species is known to be relatively thick, their large frontal sinuses make it impractical to achieve robust and accurate fixation of cannulae to the skull and it necessary to fill the frontal sinus with an alginate polymer prior to image acquisition (Rosendal et al., 2009). This adds significant time and complexity to the surgical procedure in these animals. In order to implant electrodes into the brain of Gottingen minipigs, Bjarkam et al. developed a custom-built device, head fixation device, incorporating MRI-compatible zygomatic screws and a palate tray (Bjarkam et al., 2004). However, in this study several misplaced lesions may have been caused by the placement of the fiducial markers obliquely on the skull surface.

The larger brain size of agricultural crossbred pigs such as the Large White Landrace pig compared to minipigs makes them more useful for developing drug delivery equipment of a scale that could be translated into clinical trials (Jelsing et al., 2006). However, one of the primary difficulties with using these pigs for biomedical research is their rapid rate of growth. Indeed, the average daily weight gain for Large White Landrace has been reported to be between 734 and 992 g/day in the first 6 months of life (Campbell et al., 1990). As such, between 3 and 6 months of age, these animals increase in weight from approximately 20–85 kg, making animal handling difficult. Through the development of the adjustable pig head-fixation device and stereotactic technique that are outlined in this study, many of the practical difficulties associated with the use of such large animals have been overcome.

The development of CED-based drug delivery devices in this animal model necessitated a number of compromises to be drawn. Using younger animals in these studies was an advantage due to the ease with which they could be handled and the possibility of placing an MRI coil very close to the brain. However, the very thin skulls of animals below 4 months of age, made catheter fixation to the skull difficult. In addition at this age, the white matter is inadequately myelinated thus making the target structures difficult to visualise. In contrast, animals above 6 months of age become very difficult to manage due to their large size. As such, in Large White Landrace crossbred pigs, we found that the animals can be easily managed between a window age of 4–5 months, whilst providing suitable skull thickness and brain myelination to enable catheter implantation to be performed.

It is imperative for high-precision catheter implantation that targets are clearly visible on MRI and that there is no cannula deflection on insertion. Achieving these requirements is dependent on achieving robust head immobilisation during MR imaging and burr hole drilling, as well as using cannula implantation materials with sufficient stiffness. Indeed the critical steps in the development of this cannula implantation procedure were the appreciation that adequate images could be obtained with a Sense Flex-L coil and that a rigidly held stereotactic arm (surgical robot) and effective drills, could produce a perfectly round burr hole into which the hub of an implantable catheter could push-fit.

In general, to optimise MR image quality, it is essential to maximise the signal-to-noise ratio (SNR) by having the MR coil as close to the target as possible (Fujita, 2007). The choice of MRI coil for this application was critically influenced by the need to achieve robust head immobilisation. This was particularly critical as there was a need to eliminate movement artefact being generated during MR imaging, particularly due to the animals breathing. We found that the most practical coils for imaging the brain of Large White, Landrace pigs were either a 1.5 T Sense knee coil (Philips, UK) or a 1.5 T Sense Flex-L coil (Philips, UK), which incorporates two flexible, circular elements.

In this study we have demonstrated that the use of subject-specific MRI data and stereotactic planning with ACCISS II is a far more accurate method than the use of a standardised brain atlas. In addition, we have shown that the use of pre- and post-operative MRI data can be successfully deployed as a means to determine quantitative cannula target position accuracy without the need for histology, although despite being likely, there is some uncertainty whether the volume of T1 hypointensity interpreted as the catheter-tip location is a reliable assumption. Furthermore, there is no simple strategy for precisely determining the catheter-tip position, particularly as the catheter tip has a diameter of 200 mm, which is significantly less than the MRI voxel dimensions. Overall implantation inaccuracy can be described as the single overall mean error value in each plane between the centre of the planned target sphere and the centre of the infusion. In this study, catheters designed to achieve drug delivery by CED were implanted into pig brain with a measured inaccuracy of less than mean Euclidean distance of 0.623 mm.

There are a number of important differences between this study and earlier studies developing stereotactic procedures in pigs. Early studies employed ventriculography for stereotactic targeting (Saito et al., 1998; Sakumoto et al., 2004). Ventriculography is however an invasive and technically challenging procedure that unlike MRI does not fully take into account inter-individual variations in pig brain anatomy. As a consequence Bjarkam et al. employed MR imaging using a 3 T MRI scanner to achieve highly accurate implantation of devices within subcortical structures (Bjarkam et al., 2004, 2009). However, in contrast to these studies and in common with many Neurosurgical units, we were restricted to the use of a 1.5 T rather than 3.0 T MRI scanner, leading to potentially lower resolution MR imaging. Nevertheless, through the use of an animal model without a large frontal sinus and the use of closely fitting head coils and a highly rigid robotic arm, we were able to achieve similar implantation accuracy.

The use of a robotic stereotactic arm offers a number of key advantages over stereotactic frames. Firstly, robotic arms can be used to rapidly and precisely move in and out of position and make the surgical field more accessible to the surgeon. Clearly whereas the use of a frame is constrained by the size of the subject's head, a stereotactic robotic arm can be used to target structures within the brain of animals with heads that are too large to fit within the stereotactic volume of for example the Leksell stereotactic frame used by Bjarkam et al. (2009). In addition, robotic arms provide a rigid platform for drilling to be performed. This leads to the formation of perfectly round burr holes, whereas due to subtle play in stereotactic frames the burr hole frequently becomes slightly oval. Whilst this is not especially relevant for implanting deep-brain stimulator electrodes or the temporary placement of cannulae into the brain, this can be problematic for implanting fine catheters into the brain. Specifically, with a perfectly round burr hole it was possible for us to push-fit catheter hubs into the skull securely. However, when the burr hole was slightly oval, catheter hub fixation necessitated the use of bone cement. As bone cement sets in a highly exothermic reaction this frequently led to the catheter components melting. Finally, it is likely that in future, robotic stereotactic arms will have the capability to drill burr holes and undertake catheter insertion in a fully automated manner making the procedure safer and quicker.

In summary, we have presented evidence to support the use of Large White Landrace pigs as a suitable model for the development and testing of stereotactically implantable neurosurgical equipment including cannulae and electrodes prior to translation into routine clinical practice. Through the development of a robust MRI-compatible device for robust pig head immobilisation, use of a rigid robotic stereotactic arm and ACCISS II surgical planning software, we have been able to undertake highly accurate image guided cannula delivery. This study has outlined the evolution of this stereotactic equipment and surgical procedure, as well as providing objective evidence of implantation accuracy. The target confirmation approach presented is a quantitative means to verify the implant procedure.

## Figures and Tables

**Table 1:** Relative co-ordinates of the fiducials. Sagittal co-ordinates (mm) relative to fiducial number 5. Coronal co-ordinates (mm) relative to the back of the fiducial arc. Axial co-ordinate (mm) relative to the base of the fiducial arc.

	Fiducial number								
	1	2	3	4	5	*6*	7	8	9
Sagittal	−72	−60	−36	−24	0	24	36	60	60
Coronal	99	87	111	123	135	111	123	87	75
Axial	87	104	104	77	87	104	77	87	104

**Table 2:** ACCISS-II target position co-ordinates for six subjects. Mean male porcine weight of 45 kg. Positional error for both left-hand side (LHS) and right-hand side (RHS) targets. Average Euclidean error = 0.623 mm (standev = 0.33).

Model i.d.	Target (mm)	Proposed target RHS	Positional error (mm)	Euclidean error (mm)	Proposed target LHS	Positional error (mm)	Euclidean error (mm)
MA26	*X*	111.4	0.5	0.99	89.9	0.1	0.87
	*Y*	136.9	0.7		137	0.7	
	*Z*	27.2	0.5		27.2	0.5	

MA27	*X*	87.4	0.1	0.81	112.4	0.1	0.17
	*Y*	157.7	0.1		157.3	0.1	
	*Z*	26.5	0.8		26.4	0.1	

V3	*X*	107.5	0.25	0.61	84.8	0.25	0.57
	*Y*	124.5	0.5		132.7	0.5	
	*Z*	46.7	0.25		48.7	0.1	

Thursday	*X*	111.9	0.25	1.09	91.7	0.1	0.10
	*Y*	121.2	0.7		119.8	0	
	*Z*	46.3	0.8		45.6	0	

ZR1	*X*	88.9	0.1	0.24	112	0.3	0.40
	*Y*	126.9	0.1		127.2	0.25	
	*Z*	31.3	0.2		31.3	0.1	

ZR3	*X*	112.4	0.3	0.77	89.7	0.3	0.86
	*Y*	164.1	0.5		163.4	0.8	
	*Z*	27.2	0.5		27.2	0.1	
